# Supplementary material for: Light environment drives evolution of color vision genes in butterflies and moths
Source: Commun Biol. 2021 Feb 9;4:177. doi: 10.1038/s42003-021-01688-z (PMC7873203; doi:10.1038/s42003-021-01688-z)
Supplement: Supplementary file 2 — Supplementary Information [file 42003_2021_1688_MOESM2_ESM.pdf]

Supplementary Information

to

Light environment drives evolution of color vision genes in butterflies and moths

Yash Sondhi, Emily A. Ellis, Seth M. Bybee, Jamie C. Theobald, Akito Y. Kawahara

Supplementary Figures

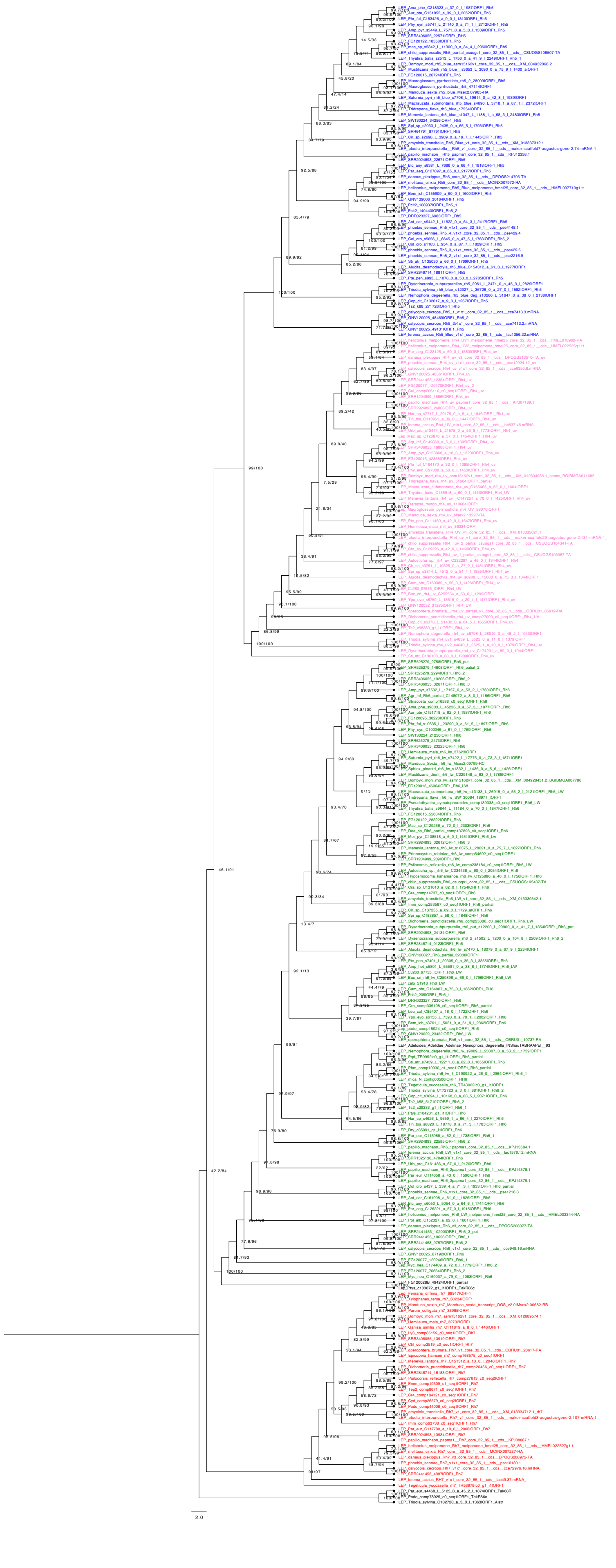
2.0

Supplementary Figure 1: Annotated nucleotide gene tree created using an alignment of 266 opsin sequences with different opsin families color coded. The tree was midpoint rooted, converted to a cladogram and ordered for ease of visualization. Numbers in parentheses are SH-aLRT support (%) / ultrafast bootstrap support (%). The final opsin annotation is based on this tree. Best Bayesian Information Criteria (BIC) score model was TIM2+F+R9 and convergence was reached after 624 iterations, an ultrafast bootstrap with 10000 iterations was run and an SH- aLRT with 1000 iterations was also performed.

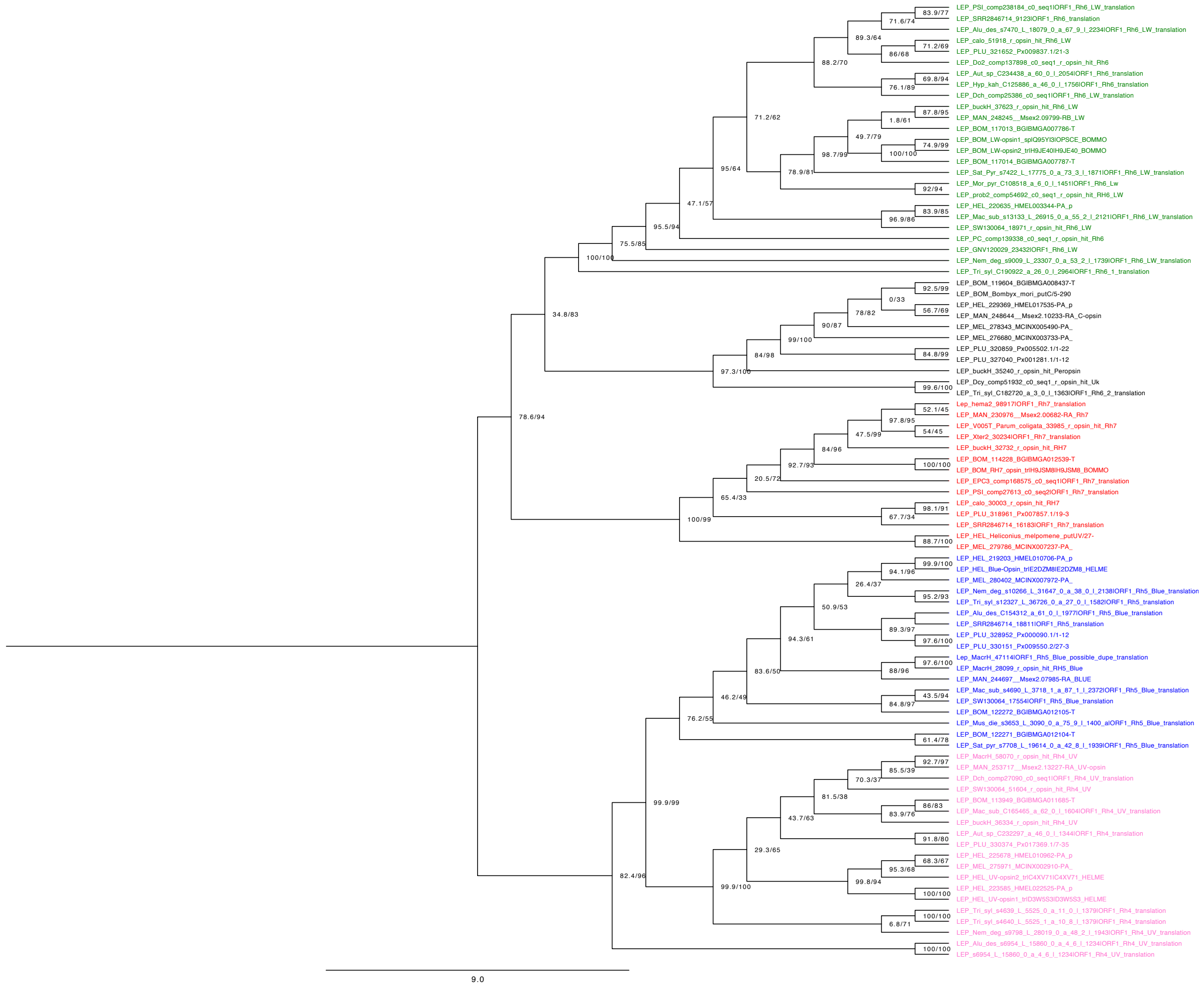

Supplementary Figure 2: Annotated amino acid gene tree using an alignment of 87 opsin sequence with different opsin families color coded. The tree was midpoint rooted, converted to a cladogram and ordered for ease of visualization. Numbers in parentheses are SH-aLRT support (%) / ultrafast bootstrap support (%). The final opsin annotation is based on this tree. Best BIC score model was LG+R5. Convergence was reached after 383 iterations. An ultrafast bootstrap with 10000 iterations was run and an SH- aLRT with 1000 iterations was also performed.

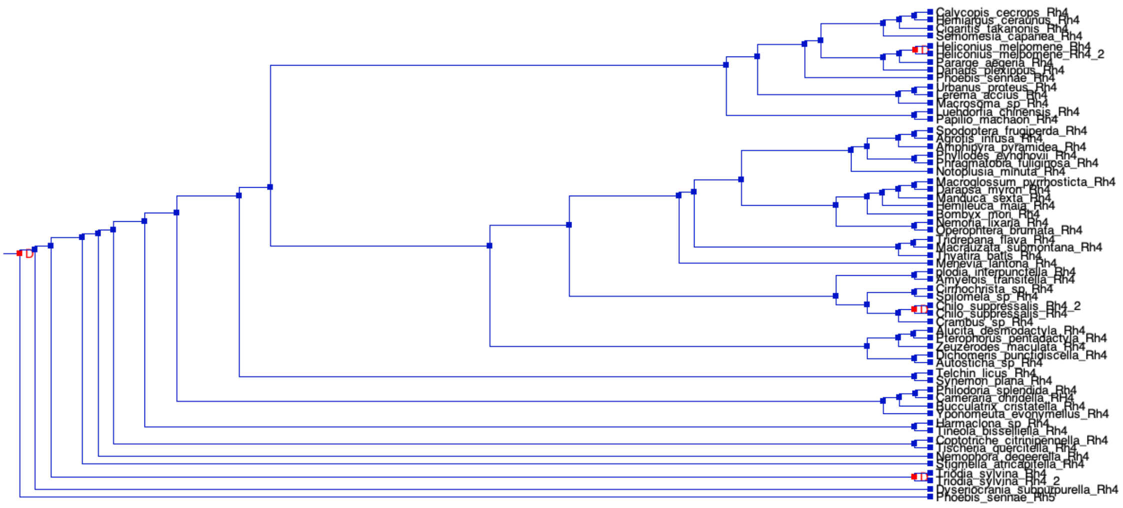

Supplementary Figure 3: UV gene reconciled tree, D represents a duplication event

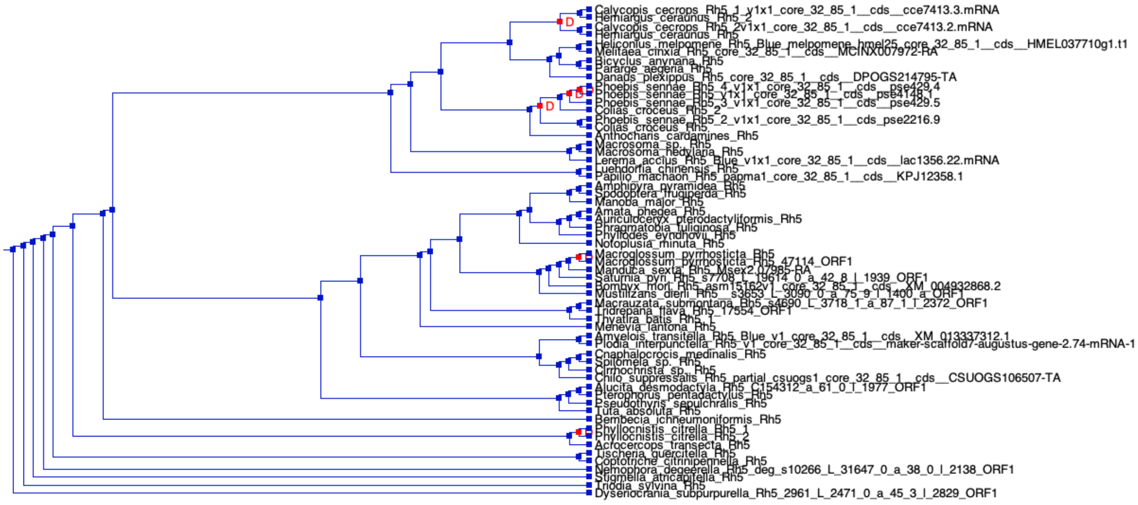

Supplementary Figure 4: Blue gene reconciled tree, D represents a duplication event

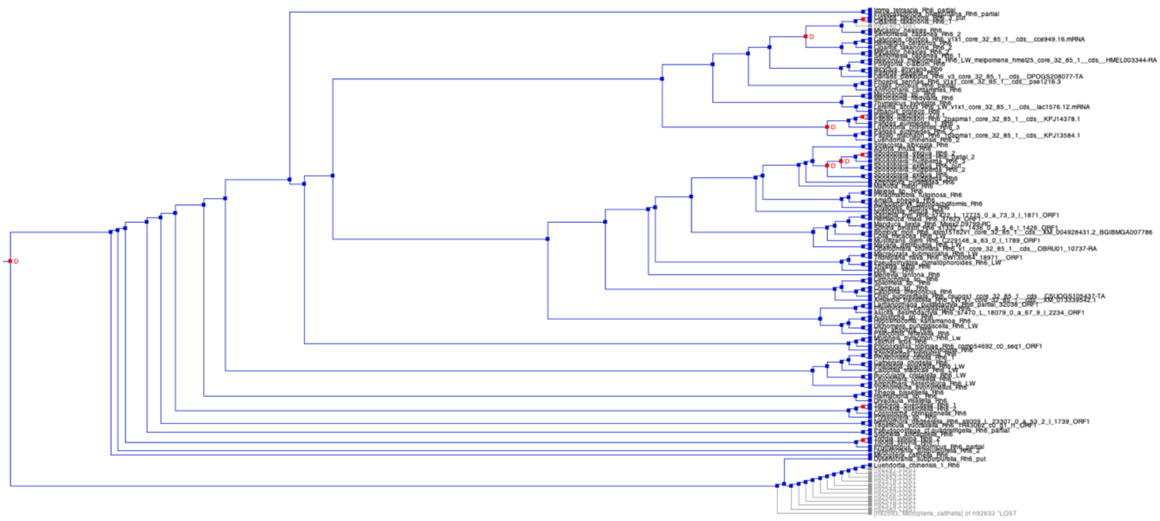

Supplementary Figure 5: LW gene reconciled tree, D represents a duplication event, greyed out lineages represent a loss. See Notung Supplementary files on Dryad for information on lineage names.

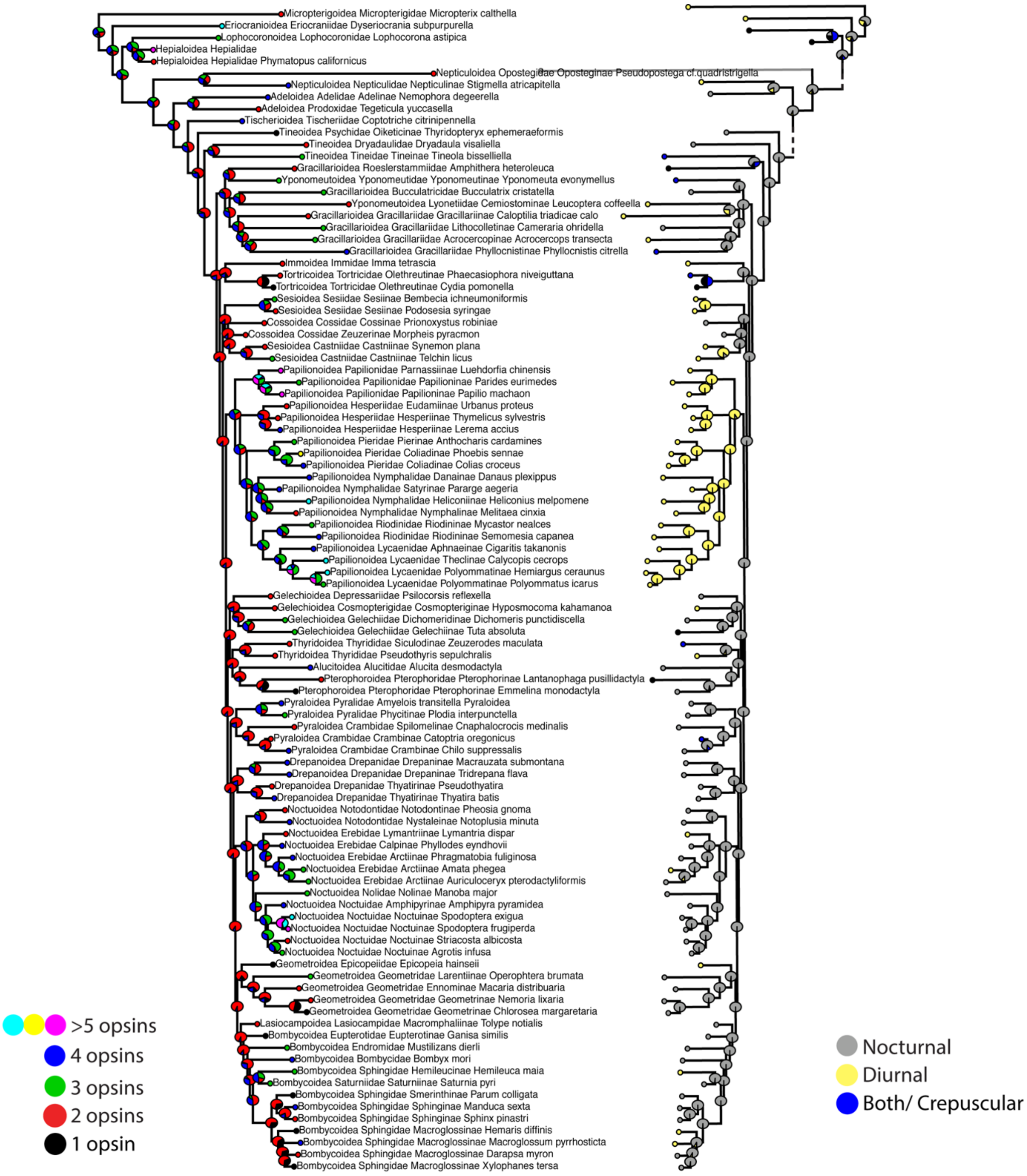

Supplementary Figure 6: Number of opsins ancestral state reconstruction plotted against ancestral state reconstruction of diel-niche.

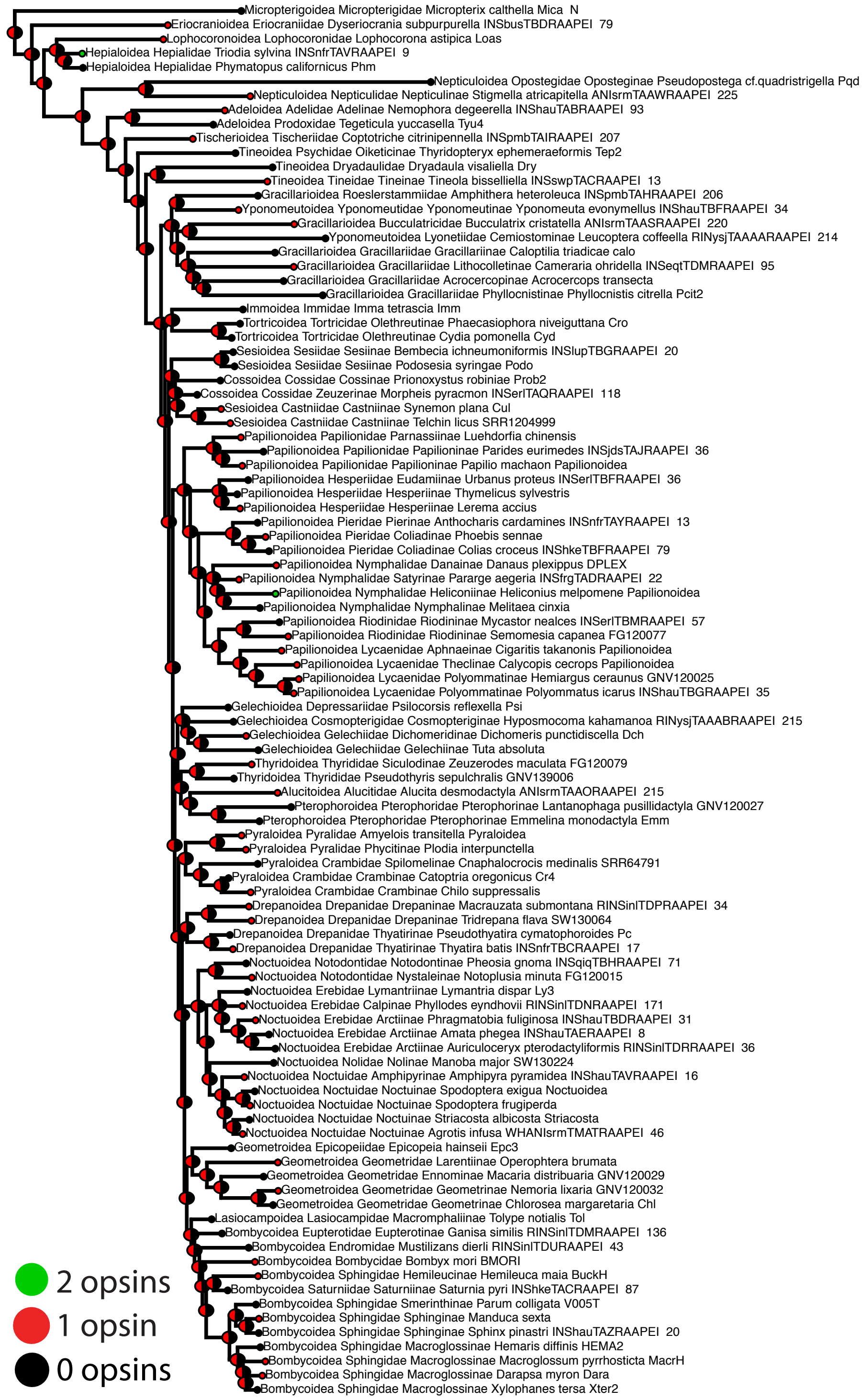

Supplementary Figure 7: UV opsin ancestral state reconstruction

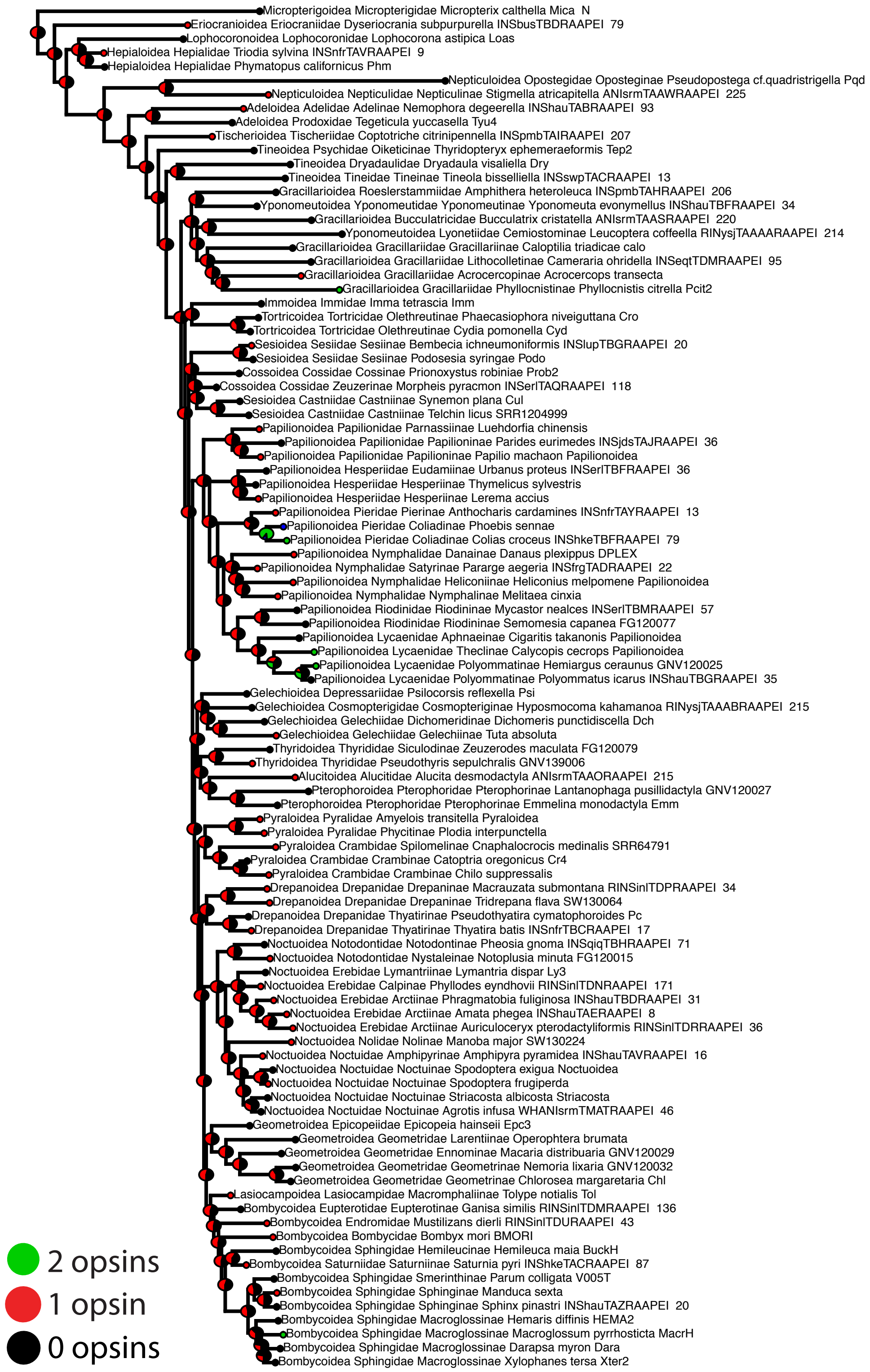

Supplementary Figure 8: Blue opsin ancestral state reconstruction

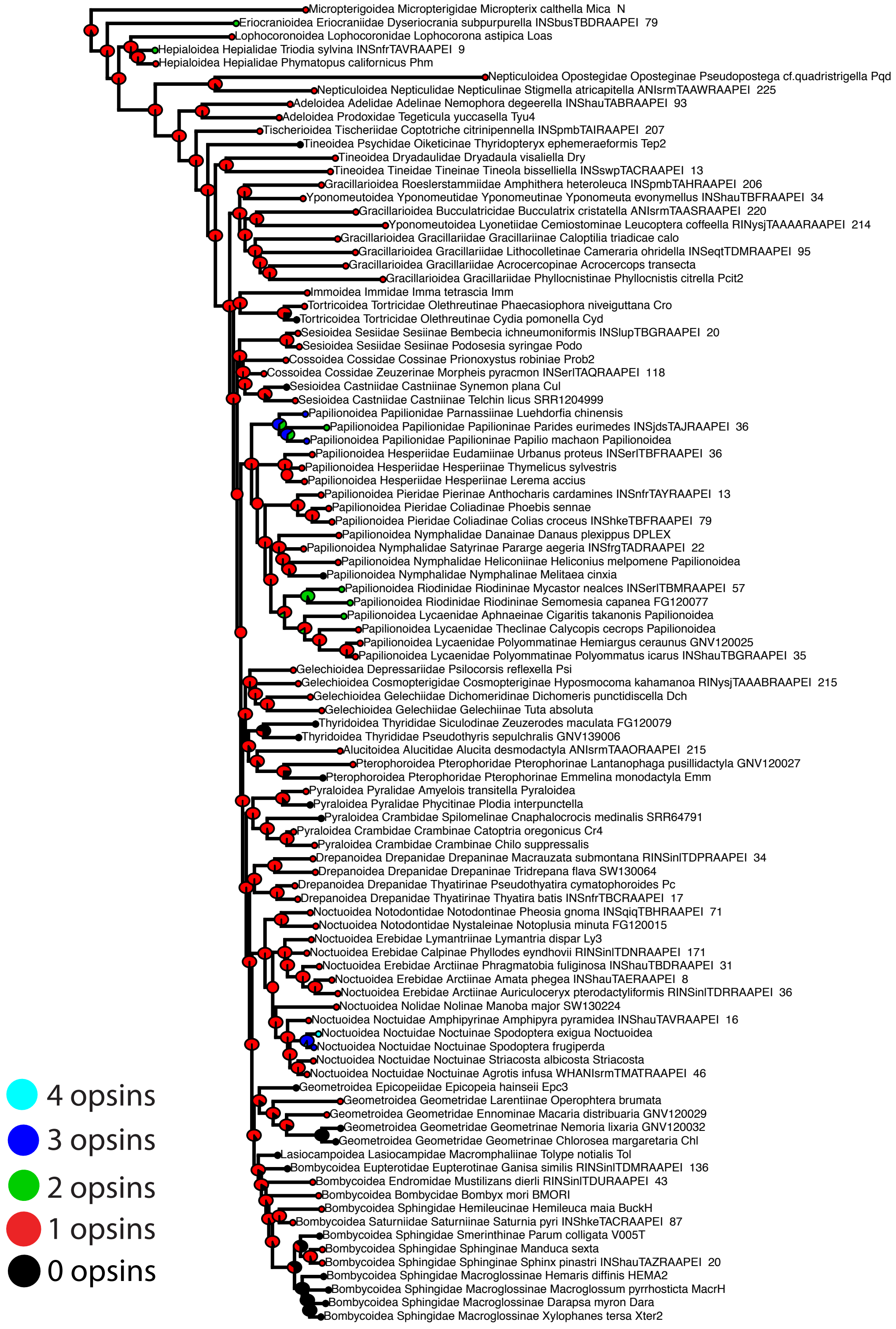

Supplementary Figure 9: LW opsin ancestral state reconstruction

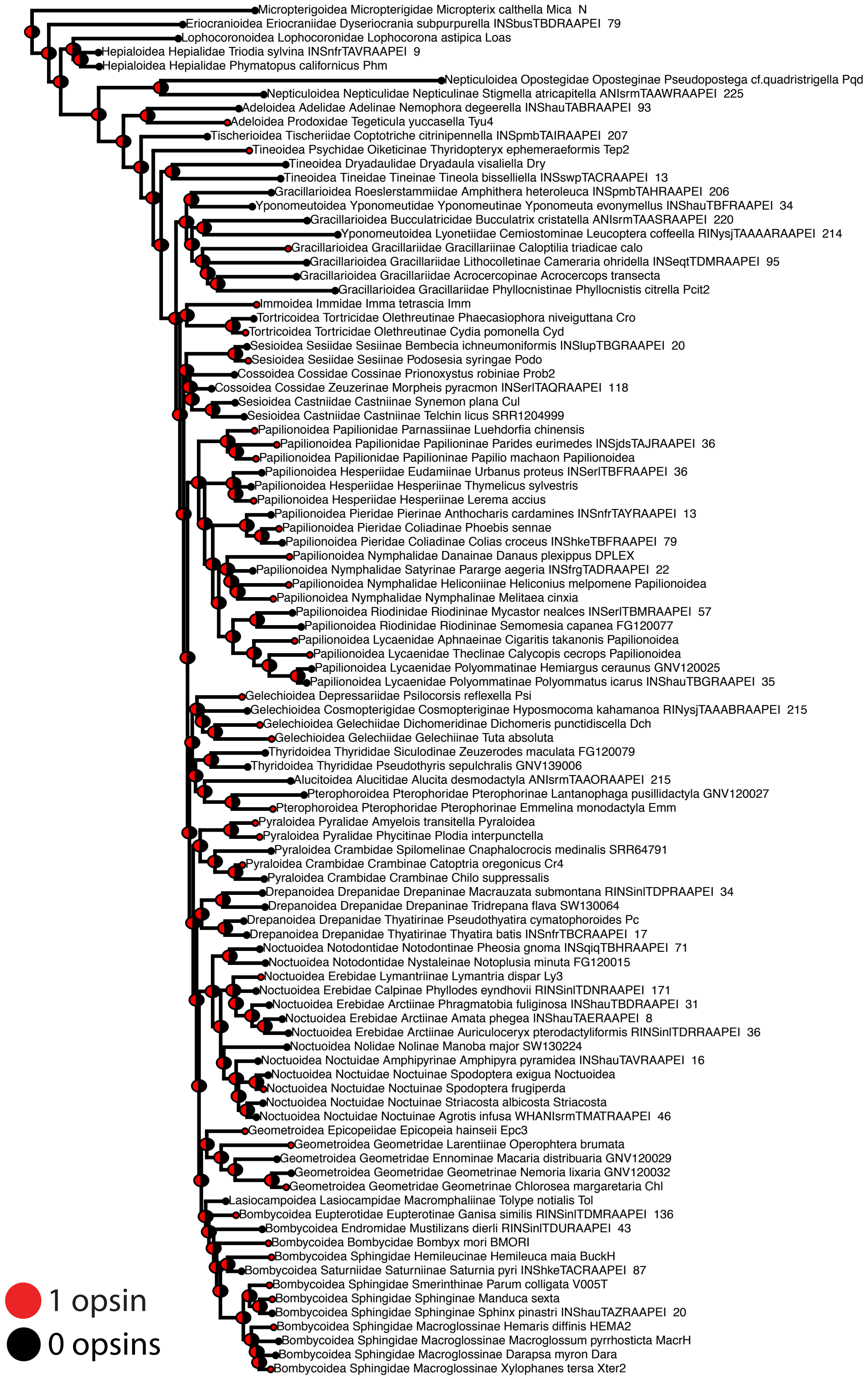

Supplementary Figure 10: RH7 opsins ancestral state reconstruction

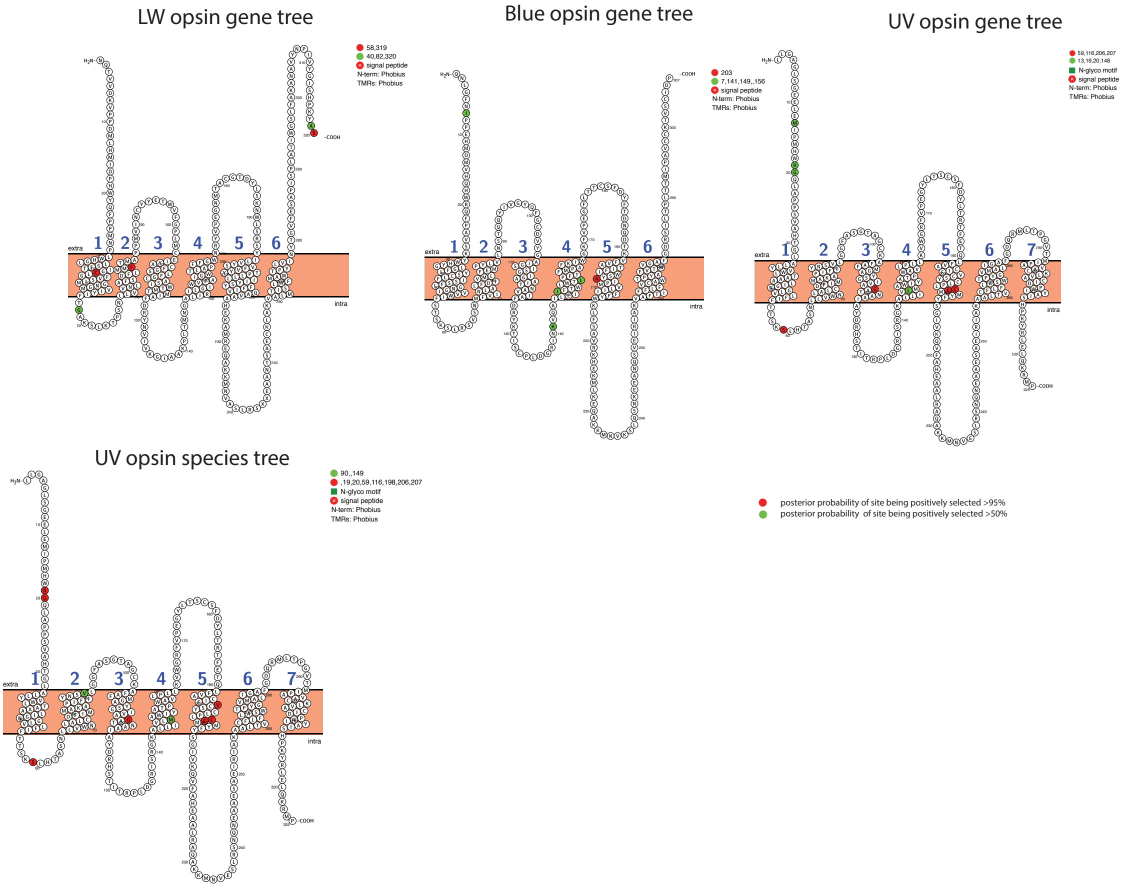

Supplementary Figure 11: Protter models for the different opsins with some opsin sites under higher dN/dS marked, see Supplementary Data 4 for an exhaustive list. Top: UV, Blue, LW gene trees. Bottom: UV opsin species trees.
