# Supplementary material for: Light environment drives evolution of color vision genes in butterflies and moths
Source: Commun Biol. 2021 Feb 9;4:177. doi: 10.1038/s42003-021-01688-z (PMC7873203; doi:10.1038/s42003-021-01688-z)
Supplement: Supplementary file 3 — Description of Supplementary Files [file 42003_2021_1688_MOESM3_ESM.pdf]

Description of Additional Supplementary Files

File name: Supplementary Data 1

Description: Lepidoptera opsins annotated with the presence and absence of opsins and the diel activity table including details on the transcriptomes as well as the source. Legend: Diel habitat N= Nocturnal, D = Diurnal, uk = Unknown, B = Both, C = Crepuscular, "?" = ambiguity in assignment, na = quality metrics not applicable Sheet2: References for diel-niche

File name: Supplementary Data 2

Description: Orthofinder results of the various transcriptomes, the original identity of the genes is taken from Munoz et al.1 The species corresponding to each assembly is listed in Data 1 (Citation for 1 : Macias-Muñoz, A., Olguin, A. G. R. & Briscoe, A. D. Evolution of Phototransduction Genes in Lepidoptera. Genome Biol. Evol. 11, 2107–2124 (2019).)

File name: Supplementary Data 3

Description: Duplication events identified by Notung

File name: Supplementary Data 4

Description: PAML and HyPhy implementations of selection models of differentdatasets, ** refer to p-value <0.01, * refers top-value <0.1, bold text refers to sites in common across different selection analyses.
