# Supplementary material for: Light environment drives evolution of color vision genes in butterflies and moths
Source: Commun Biol. 2021 Feb 9;4:177. doi: 10.1038/s42003-021-01688-z (PMC7873203; doi:10.1038/s42003-021-01688-z)
Supplement: Supplementary file 8 — Reporting Summary [file 42003_2021_1688_MOESM8_ESM.pdf]

nature research

Corresponding author(s): Yash Sondhi

Last updated by author(s): Dec 22, 2020

Reporting Summary

Nature Research wishes to improve the reproducibility of the work that we publish. This form provides structure for consistency and transparency in reporting. For further information on Nature Research policies, see Authors & Referees and the Editorial Policy Checklist.

Statistics

For all statistical analyses, confirm that the following items are present in the figure legend, table legend, main text, or Methods section.

n/a	Confirmed	
☐	☒	The exact sample size (n) for each experimental group/condition, given as a discrete number and unit of measurement
☒	☐	A statement on whether measurements were taken from distinct samples or whether the same sample was measured repeatedly
☐	☒	The statistical test(s) used AND whether they are one- or two-sided<br>*Only common tests should be described solely by name; describe more complex techniques in the Methods section.*
☒	☐	A description of all covariates tested
☒	☐	A description of any assumptions or corrections, such as tests of normality and adjustment for multiple comparisons
☒	☐	A full description of the statistical parameters including central tendency (e.g. means) or other basic estimates (e.g. regression coefficient) AND variation (e.g. standard deviation) or associated estimates of uncertainty (e.g. confidence intervals)
☐	☒	For null hypothesis testing, the test statistic (e.g. F, t, r) with confidence intervals, effect sizes, degrees of freedom and P value noted<br>*Give P values as exact values whenever suitable.*
☐	☒	For Bayesian analysis, information on the choice of priors and Markov chain Monte Carlo settings
☐	☒	For hierarchical and complex designs, identification of the appropriate level for tests and full reporting of outcomes
☒	☐	Estimates of effect sizes (e.g. Cohen's d, Pearson's r), indicating how they were calculated

Our web collection on statistics for biologists contains articles on many of the points above.

Software and code

Policy information about availability of computer code

Data collection	Custom python scripts provided for data filtration and processing, scripts available as supplementary information
Data analysis	PIA(https://github.com/xibalbanus/PIA2) MAFFT (v7.294b) IQ-TREE (multi-core v1.6.12) Archaeopteryx (v0.9917 beta)(https://github.com/cmzmasek/archaeopteryx-js) Geneious v. 10.0.9 AliView v. 1.18.1 Muscle v. 3.8.31 RStudio Version 1.2.1335 R 3.5 R packages: ape v. 5.397, phytools,geiger Notung v2.9.15 Orthofinder v.2.3.11 Ete3 Python 2,3 Ete3 Protter (http://wlab.ethz.ch/protter/) Swisspdb viewer PAML 4.9a TranslatorX

For manuscripts utilizing custom algorithms or software that are central to the research but not yet described in published literature, software must be made available to editors/reviewers. We strongly encourage code deposition in a community repository (e.g. GitHub). See the Nature Research guidelines for submitting code & software for further information.

Data

Policy information about availability of data

All manuscripts must include a data availability statement. This statement should provide the following information, where applicable:

- Accession codes, unique identifiers, or web links for publicly available datasets
- A list of figures that have associated raw data
- A description of any restrictions on data availability

The authors declare that [the/all other] data supporting the findings of this study are available within the paper [and its supplementary information files]. The data is presently available on Dropbox and will be uploaded to Dryad.

Field-specific reporting

Please select the one below that is the best fit for your research. If you are not sure, read the appropriate sections before making your selection.

☐ Life sciences ☐ Behavioural & social sciences ☒ Ecological, evolutionary & environmental sciences

For a reference copy of the document with all sections, see nature.com/documents/nr-reporting-summary-flat.pdf

Ecological, evolutionary & environmental sciences study design

All studies must disclose on these points even when the disclosure is negative.

Study description	We used genes mined from available transcriptomes and analysed them in the context of a pruned phylogenies.
Research sample	The research sample was mined opsins from publicly available data along with natural history data collected from literature and published phylogenies
Sampling strategy	We tested as many samples as could recover and allign an opsin without creating a gap filled alignment.
Data collection	Data was collected for diel-niche by analyzing literature and communicating with other experts. The molecular data was adapted from published transcriptomes and phylogenies and was mined using scripts available in the manuscript.
Timing and spatial scale	We used published data, or previously assembled data.
Data exclusions	Data was excluded where mining returned no opsins.
Reproducibility	Analysis were repeated varying the parameters of the models and by filtering out uncertain data points.
Randomization	We wanted to cover as broad a sample as possible so randomization was not required.
Blinding	No need for blinding.

Did the study involve field work? ☐ Yes ☒ No

Reporting for specific materials, systems and methods

We require information from authors about some types of materials, experimental systems and methods used in many studies. Here, indicate whether each material, system or method listed is relevant to your study. If you are not sure if a list item applies to your research, read the appropriate section before selecting a response.

Materials & experimental systems

n/a	Involved in the study	
☒	☐	Antibodies
☒	☐	Eukaryotic cell lines
☒	☐	Palaeontology
☒	☐	Animals and other organisms
☒	☐	Human research participants
☒	☐	Clinical data

Methods

n/a	Involved in the study	
☒	☐	ChIP-seq
☒	☐	Flow cytometry
☒	☐	MRI-based neuroimaging
